# Mutual learning with memory for semi-supervised pest detection

**DOI:** 10.3389/fpls.2024.1369696

**Published:** 2024-06-17

**Authors:** Jiale Zhou, He Huang, Youqiang Sun, Jiqing Chu, Wei Zhang, Fang Qu, Huamin Yang

**Affiliations:** ^1^ Science Island Branch, Graduate School of USTC, Hefei, China; ^2^ Institute of Intelligent Machines, Hefei Institute of Physical Science, Chinese Academy of Sciences, Hefei, China; ^3^ Technology Research and Development Center, Anhui Zhongke Intelligent Sense Industrial Technology Research Institute, Wuhu, China

**Keywords:** semi-supervised pest detection, mutual learning, memory fusion, Spatial-aware Multi-Resolution Feature Extraction, cascade RPN

## Abstract

Effectively monitoring pest-infested areas by computer vision is essential in precision agriculture in order to minimize yield losses and create early scientific preventative solutions. However, the scale variation, complex background, and dense distribution of pests bring challenges to accurate detection when utilizing vision technology. Simultaneously, supervised learning-based object detection heavily depends on abundant labeled data, which poses practical difficulties. To overcome these obstacles, in this paper, we put forward innovative semi-supervised pest detection, PestTeacher. The framework effectively mitigates the issues of confirmation bias and instability among detection results across different iterations. To address the issue of leakage caused by the weak features of pests, we propose the Spatial-aware Multi-Resolution Feature Extraction (SMFE) module. Furthermore, we introduce a Region Proposal Network (RPN) module with a cascading architecture. This module is specifically designed to generate higher-quality anchors, which are crucial for accurate object detection. We evaluated the performance of our method on two datasets: the corn borer dataset and the Pest24 dataset. The corn borer dataset encompasses data from various corn growth cycles, while the Pest24 dataset is a large-scale, multi-pest image dataset consisting of 24 classes and 25k images. Experimental results demonstrate that the enhanced model achieves approximately 80% effectiveness with only 20% of the training set supervised in both the corn borer dataset and Pest24 dataset. Compared to the baseline model SoftTeacher, our model improves *mAP*_@0.5_ (mean Average Precision) at 7.3 compared to that of SoftTeacher at 4.6. This method offers theoretical research and technical references for automated pest identification and management.

## 1 Introduction

Many problems hinder the development of agriculture, such as climatic conditions, soil quality, pests, and diseases. Among them, crop pests are a very important problem that has a serious negative impact on agricultural output. For farmers, crop pest management has long been a top concern because it is essential for ensuring global food security and steady economic growth. In traditional agriculture, agricultural professionals are needed for monitoring responsibilities. However, there are a number of shortcomings with manual investigations, including limited efficiency, subjectivity, and error proneness. The development of a highly accurate and efficient automatic pest monitoring system is desirable for food security and productivity.

Thankfully, as information science advances, new approaches to problem-solving are presented (Li et al., 2021). One such approach is precision agriculture (Khan and AlSuwaidan, 2022), which combines information technology and agricultural output. The two primary aspects of early machine learning framework research, as seen from an algorithmic perspective, are the extraction of pest-related information from photos as feature vectors and the use of machine learning classifiers for categorization. In order to accurately identify and categorize cotton crop diseases, Camargo and Smith (2009) retrieved picture features from regions afflicted by the diseases, kept only the most important features, and fed the Support Vector Machine with the feature set. In order to create a multi-class classifier for the identification of 24 pest classes, Xie et al. (2015) employed a sparse-coding histogram with several feature modalities to represent pest images. Several-kernel learning (SKL) approaches were then utilized to fuse numerous features. The spectral residual (SR) approach was used by Qin et al. (2019) to extract edge characteristics from stored-grain pests, and these features are then used for saliency edge detection. The success of the previously described models (Camargo and Smith, 2009; Xie et al., 2015; Qin et al., 2019; Li et al., 2021), which were based on classical machine learning, was largely dependent on the controllability of external environmental elements and the correctness of manually derived characteristics from target regions.

Deep learning has become widely used in agriculture as a result of its recent quick advancement that has outpaced standard machine learning techniques. In order to automatically detect and count pests, Ding and Taylor (2016) proposed a sliding-window detection technique in 2016. This approach included a convolutional neural network. A generative adversarial network with numerous attention, residual, and dense fusion methods was proposed by Dai et al. (2020) to upscale low-quality pest photos, therefore improving spatial resolution and recovering high-frequency details. The recall rate for pest identification was greatly increased by this method. In order to improve the characteristics of small-object pest regions, Wang et al. (2021) proposed a sampling-balanced region proposal network and integrated an attention mechanism into the residual network. In addition to electronic traps for monitoring, Huang et al. (2021) presented the Multi-Attention and Multi-Part Convolutional Neural Network (MAMPNet) for citrus fly identification. Apart from network improvements, data-related aspects have also been investigated by researchers (Cubuk et al., 2019; Yamada et al., 2019; Cubuk et al., 2020). In order to obtain distinct multi-scale representations, Li et al. (2019) proposed an effective data augmentation strategy for algorithms based on a convolutional neural network (CNN). This strategy entailed rotating images at different degrees and cropping them to different grids during training. The strategy’s efficacy across four pest datasets was finally demonstrated by fusing detection findings from various scale photos. However, these approaches (Ding and Taylor, 2016; Dai et al., 2020) mostly depend on the manual annotation of large amounts of data for every kind of pest, necessitating the training datasets to have bounding boxes identified. Manually labeling a large amount of data consumes many manpower and material resources, which brings trouble to the practical application of detection technology. Traditional target detection relies heavily on manual annotation of large amounts of data for each pest, and manual annotation of large amounts of data is time-consuming and labor-intensive.

**Figure 1:**
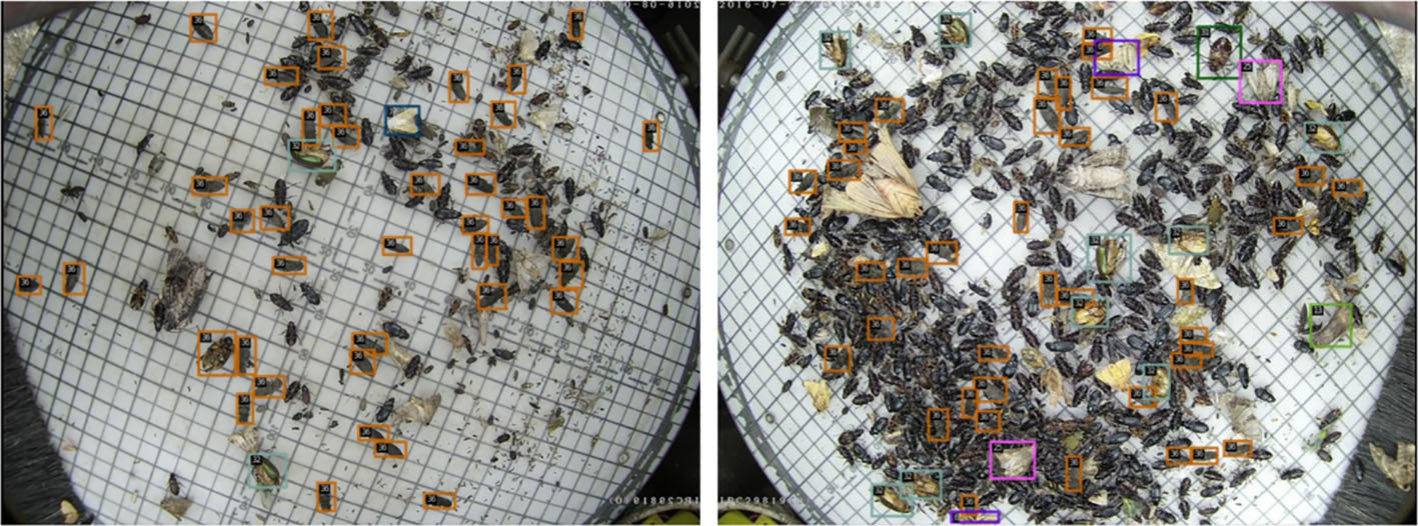
Some examples of pest images in Pest24. The picture shows how time-consuming and laborious manual labeling of data is.

Manually labeling the pictures required for pest detection requires labeling of categories and selecting the area where the pests are located, which is time-consuming and labor-intensive, as shown in **Figure 1**. Usually, it takes approximately 6 seconds to mark a box. On average, there are more than 10 pests in one pest image, so it takes 1 minute to mark one image. For example, the Pest24 dataset used in this article has 12,701 training images, 5,077 verification images, and 7,600 test images. It takes 25,378 minutes to annotate these data. The semi-supervised object detector only needs a small part of the training set of the detector based on supervised learning to achieve similar effects to supervised learning.

Innovations in semi-supervised object identification provide creative answers to these problems. Semi-supervised object detection techniques come in two types: consistency constraint-based approaches (Jeong et al., 2019) strengthen the robustness of the model’s feature extraction by combining data pairs with the original images, applying weak data augmentation to unlabeled data, and enforcing consistency constraints on the model outputs. The other kind of model relies on self-learning (Sohn et al., 2020; Xu et al., 2021; Zhou et al., 2021), in which labeled data are used to learn a pre-trained model. After that, the model makes predictions on unlabeled data and uses confidence threshold filtering and post-processing to create pseudo-labels. To improve overall performance, the model is then trained using both the original labeled data and the created pseudo-labels. A pseudo-label-based technique was presented by Self-Training and Augmentation driven Consistency regularization (STAC) (Sohn et al., 2020), which was a noteworthy advancement in the field of self-supervised semi-supervised detection. Instant-Teaching (Zhou et al., 2021) is a proposed online pseudo-label generation method. Unlike STAC, which generates pseudo-labels only once throughout the entire process without updating them during training, Instant-Teaching uses offline generation. An end-to-end pseudo-label-based semi-supervised object identification network, called SoftTeacher (Xu et al., 2021), was introduced along with two simple yet powerful methods to choose dependable pseudo-boxes for learning box regression: the box jitter method and the soft teacher mechanism.

However, traditional semi-supervised networks still face numerous challenges. It is important to address the discrepancies among the detection results in the same image that occur during different training iterations. Confirmation bias (Tarvainen and Valpola, 2017) is a common problem in semi-supervised learning. When the model generates incorrect predictions with high confidence, these incorrect predictions will be further reinforced through incorrect pseudo annotations. In other words, the model itself struggles to rectify these false predictions. Furthermore, the research subject of pests being small targets has resulted in numerous challenges. One such challenge is the limited availability of features. Due to the small size of objects, as the number of CNN layers increases during feature extraction, the target feature information tends to be progressively weakened, making it difficult to extract discriminative features. Moreover, in the context of a multi-layer network, this issue can also lead to missed detections of certain objects. Another challenge is the high positioning accuracy requirements. Compared to objects of typical size, small targets like pests pose a challenge for the Region Proposal Network (RPN) in generating effective candidate target regions. This, in turn, makes it difficult to accurately regress on the proposed boxes during subsequent stages. The main contributions of this paper are as follows:

1) We introduce PestTeacher, an innovative semi-supervised pest detection framework that effectively mitigates the issues of confirmation bias and instability among detection results across different iterations. Our framework enhances the quality of pseudo annotations through mutual learning with memory scheme, resulting in improved object detection performance.
2) We propose Spatial-aware Multi-Resolution Feature Extraction module to address small target feature information that tends to be weakened as the number of CNN layers increases. The Cascade RPN module (Vu et al., 2019) is capable of generating higher-quality anchors.
3) To provide a comprehensive evaluation of the improved model, we conducted extensive experiments using data from both the Pest24 dataset and the corn borer dataset. The experimental results demonstrate that our approach achieves superior performance and robustness.

## 2 Methodologies

### 2.1 Dataset

**Figure 2:**
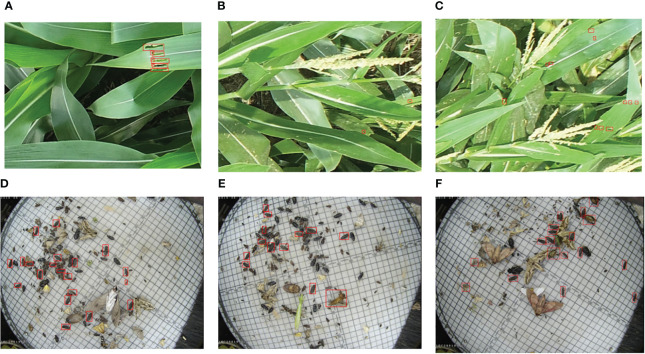
Some examples of pest images in corn borer and Pest24; the pests and pest regions that require prediction are indicated by the red boxes. **(A–C)** Images from the corn borer dataset containing various stages of corn development. **(D–F)** Images from the Pest24 dataset exhibit features like overlap and dense dispersion.

We conducted our experiments on two benchmark datasets, the corn borer pest region dataset and the Pest24 dataset (Wang et al., 2020), as shown in **Figure 2**. We collected the corn borer pest region dataset in the demonstration area of an unmanned farm in Bozhou City, Anhui Province, China, using DJI Spirit 4RTK UAV. DJI Spirit 4 RTK is a small multi-rotor high-precision aerial survey drone with a centimeter-level navigation and positioning system and a high-performance imaging system. In contrast, we collected the Pest24 dataset using the professional automatic pest image acquisition equipment developed by the Institute of Intelligent Machines, Chinese Academy of Sciences. The corn borer dataset encompasses data from various corn growth cycles. The Pest24 dataset is a large-scale, multi-pest image dataset consisting of 24 classes and 25k images, as shown in **Table 1**. The corn borer pest region dataset consists of a total of 1,424 valid samples with a resolution of 4864 × 3648. Among these samples, 502 belong to the V12 stage and are named DV12, while 922 belong to the VT stage and are named DVT. The V12 stage is shown in **Figure 2A**, with more corn leaves and a relatively pure background. In the VT stage, shown in **Figures 2B, C**, the occurrence of corn pollination, corn earing, and other phenomena will produce a more complex image background at this stage. We randomly divided DV12 and DVT into the training, validation, and test sets in an 8:1:1 ratio. We then expanded DV12 and DVT using data augmentation techniques, resulting in 1,720 and 3,217 images, respectively. We combined them to form a total of 4,937 samples for model training.

**Table 1:** Description of the 24 classes of pests in Pest24.

Index	Pest name	Relative scale	Color discrepancy
1	Rice planthopper	0.034	57.78
2	Rice leaf roller	0.123	79.55
3	Striped rice borer	0.186	74.48
5	Armyworm	0.394	91.97
6	Bollworm	0.281	87.84
7	Mcadow borer	0.226	73.08
8	*Athetis lepigone*	0.13	89.65
10	*Spodoptera litura*	0.458	89.16
11	*Spodoptera exigua*	0.138	83.44
12	Stem borer	0.277	78.69
13	Little Gecko	0.57	110.52
14	*Plutella xylostella*	0.043	83.03
37	Melahotus	0.158	188.86
15	Spodoptera cabbage	0.42	106.05
16	*Scotogramma trifolii* Rottemberg	0.28	91.63
24	Yellow tiger	0.398	90.48
25	Land tiger	0.639	98.3
28	Eight-character tiger	0.441	119.98
29	*Holotrichia oblita*	0.334	221.38
31	*Holotrichia parallela*	0.255	189.16
32	*Anomala corpulenta*	0.249	164.35
34	*Gryllotalpa orientalis*	0.95	139.06
35	Nematode trench	0.32	143.15
36	*Agriotes fuscicollis* Miwa	0.114	173.86
37	Melahotus	0.158	188.86

The Pest24 dataset comprises 25,378 multi-pest images with a resolution of 800 × 600 pixels. It includes 24 categories, which feature ultra-small object sizes, dense object distributions, high similarity among pest objects in terms of shape and color, and numerous object adhesions in the images.

### 2.2 Flowchart of framework

We present an overview of the pseudo-labeling framework for semi-supervised object detection. In the first stage, the Teacher1 model is trained on labeled data. The second stage is to perform semi-supervised training on labeled and unlabeled data. During semi-supervised training, we initialize the Teacher1 model using the pre-trained parameters. Then, we apply weak augmentation to unlabeled images and feed them into both the Teacher1 and Teacher2 models to generate pseudo-labels. To improve the quality of pseudo-labels and stabilize the semi-supervised training process, we use non-maximum suppression (NMS) to fuse the latest prediction results of the Teacher1 model, the recent detection results of the Teacher2 model, and the historical pseudo-labels generated by the Teacher2 model.

**Figure 3:**
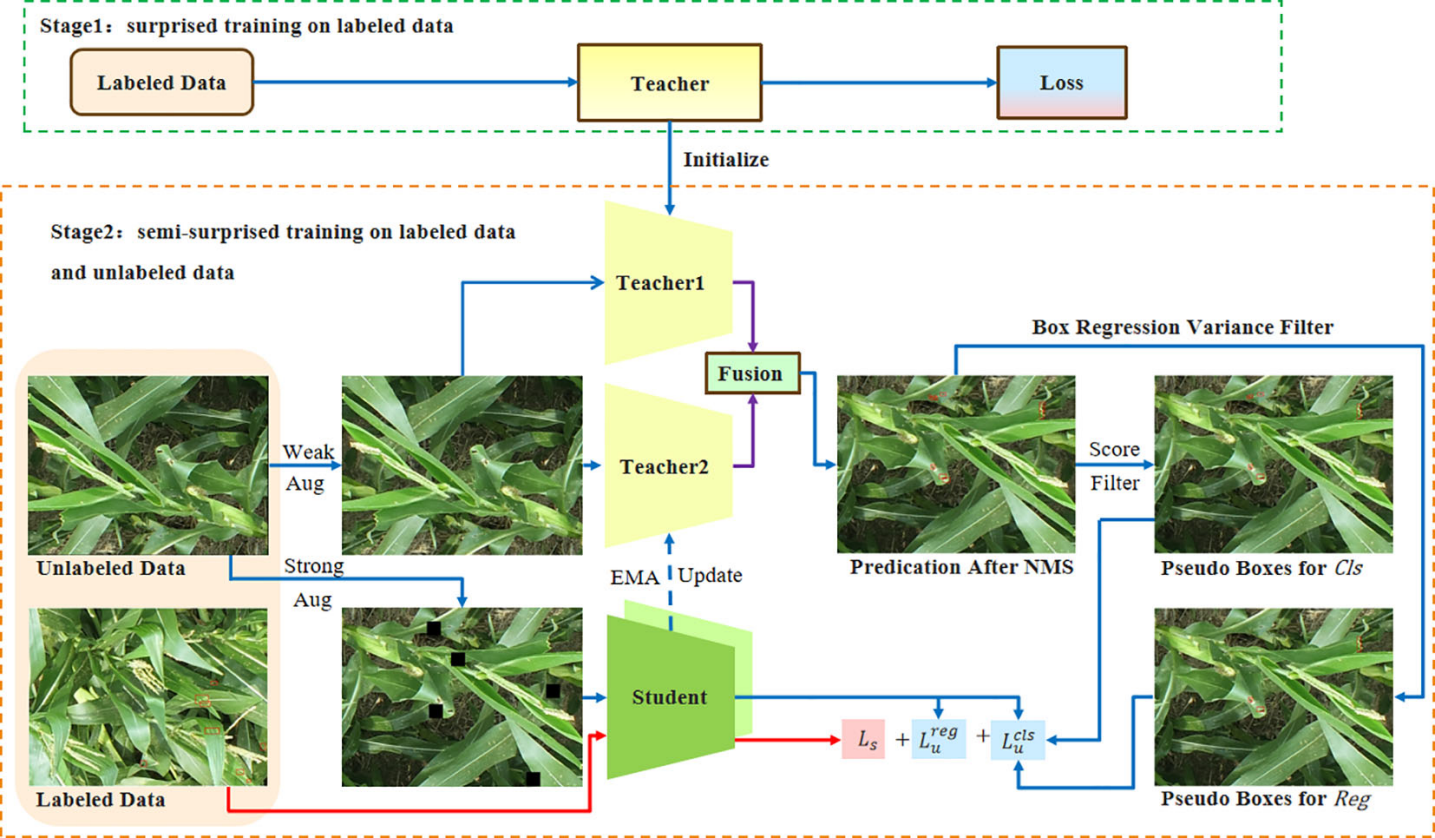
The figure shows the flowchart of framework. The training of student model uses labeled images and pseudo-labels. The pseudo-label is obtained by inferring the weak augmentation image from the teacher model. After training, the student model is used to update the teacher model using the EMA method. EMA, exponential moving average.

The student model is trained using both the detection losses on labeled images and the pseudo boxes on unlabeled images. The unlabeled images have two sets of pseudo boxes, which are used to drive the training of the classification branch and the regression branch. The Teacher2 model is an exponential moving average (EMA) of the student model. Within this framework, we have incorporated three crucial designs: mutual learning with memory, Spatial-aware Multi-Resolution Feature Extraction, and Cascade RPN. The flowchart of the framework of our network is shown in **Figure 3**.

### 2.3 Mutual learning with memory

**Figure 4:**
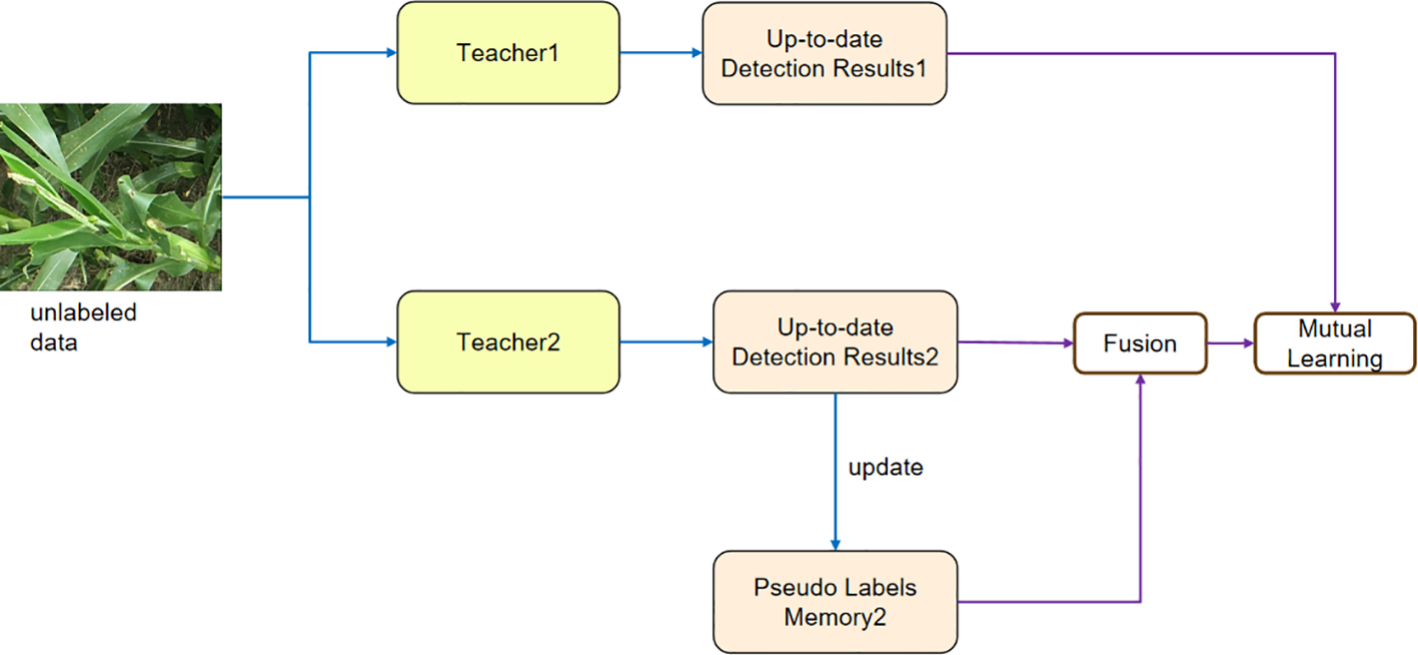
The figure shows the structure of mutual learning with memory, which alleviates the instability among the detection results in different iterations and confirmation bias.

To address the confirmation bias problem and discrepancies among the detection results, we propose mutual learning with memory mechanism, as depicted in **Figure 4**. This mechanism involves feeding weakly augmented unlabeled data into both the Teacher1 and Teacher2 models. The Teacher1 model is obtained through training with labeled data. The pseudo-label generated by the Teacher2 model is then fused with the pseudo-label stored in memory. Finally, the fused pseudo-label, along with the pseudo-label generated by the Teacher1 model, is fed into the mutual learning module.

#### 2.3.1 Mutual learning

Confirmation bias (Tarvainen and Valpola, 2017) is a prevalent issue in semi-supervised learning, where incorrect predictions with high confidence can be further reinforced through incorrect pseudo annotations. Consequently, rectifying these false predictions becomes challenging for the model itself.

To alleviate this problem, we propose a mutual learning scheme, which trains two models *t*_1_ and *t*_2_. *t*_1_ is trained from labeled data. These two models help each other to rectify the false predictions, as shown in **Figure 4**.

We take model *t*_1_ as an example, and the rectified pseudo annotations of model *t*_2_ are constructed in a similar way. When generating pseudo annotations during each training iteration, models *t*_1_ and *t*_2_ first predict class probabilities $c_{i},c_{i}^{r}$ and bounding box coordinates $b_{i},b_{i}^{r}$ on the weakly augmented unlabeled image. Finally, the rectified class probabilities and bounding box coordinates are the weighted average of $c_{i},c_{i}^{r}$ and $b_{i},b_{i}^{r}$ , where *e*_1_ gradually decreases and *e*_2_ gradually increases with training. At the beginning of training, the quality of pseudo-labels generated by Teacher1 trained with partially labeled data is significantly higher than that of the Teacher2 model, but the Teacher2 model gradually surpasses the effect of Teacher1.

The multual learning process can be expressed as Equation 1:


(1) $$\left\{ \begin{array}{l} (c_{i},{\text{b}}_{i})=t_{1}({\text{x}}_{u}),\\ (c_{i}^{r},{\text{b}}_{i}^{r})=t_{2}({\text{x}}_{u}),\\ \;c_{i}^{*}\;=e_{1}c_{i}+e_{2}c_{i}^{r},\\ \;{\text{b}}_{i}^{*}\;=\frac{1}{e_{1}c_{i}+e_{2}c_{i}^{r}}({\text{b}}_{i}e_{1}c_{i}+{\text{b}}_{i}^{r}e_{2}c_{i}^{r}), \end{array}\right.$$

#### 2.3.2 Memory fusion

**Figure 5:**
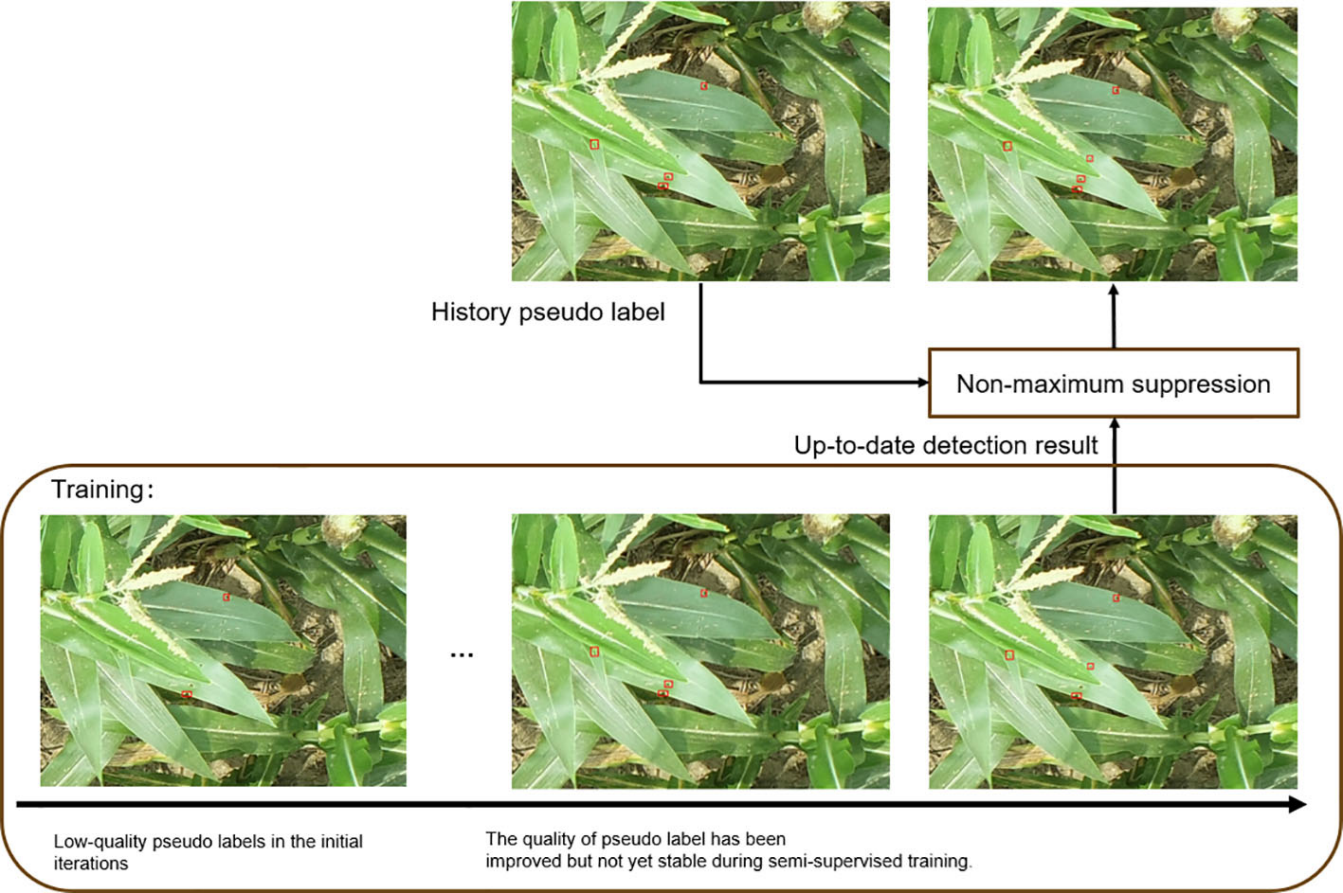
Illustration of pseudo-label fusion. The pseudo-label in semi-supervised learning is crafted by merging the latest detection outcomes with the historical pseudo-label. This fusion enhances the overall quality and stability of pseudo-labels, contributing to improved convergence during the training process.

As shown in **Figure 5**, the predicted detection results from different iterations are different. Therefore, the training procedure might become unstable and encounter difficulties with convergence if we were to use these unstable results directly as pseudo-labels on unlabeled data. Still, the results of various iterations contain a variety of knowledge.

As a result, creating an ensemble with these outputs would improve the pseudo-label quality. To achieve this, we propose employing NMS to fuse these outputs, as illustrated in **Figure 5**. We use NMS to retain the area that most likely represents the real target when multiple overlapping targets or areas are detected and to eliminate redundant detections in the overlapping areas. During semi-supervised training, this method seeks to smooth the detection results and take advantage of the differences in the outputs from various iterations. To be more precise, we use the pre-trained model to estimate each unlabeled case’s detection results, which are then saved in memory. Recently, networks with memory have been introduced, which enhance the learning and reasoning capabilities of deep learning models. This is accomplished using memory to store prior information and efficiently model the dataset’s data distribution (Chen et al., 2018; Wu et al., 2018; Yang et al., 2019). A feature embedding for every image was stored using earlier techniques (Chen et al., 2018; Wu et al., 2018), and it was updated using an exponential moving average. In contrast, our method stores the detection result and updates it through the application of NMS. Specifically, let $\left\{\bar{\text{p}},\bar{\text{t}}\right\}$ represent the stored predicted detection result of an image in pseudo-label memory, and let {p,t} be the up-to-date prediction result from the network during semi-supervised training. Then, the updating process can be expressed as Equation 2:


(2) $$\left\{\hat{\text{p}},\hat{\text{t}}\right\}=\text{NMS }(\text{CAT }(\left\{\bar{\text{p}},\bar{\text{t}}\right\},\left\{\text{p},\text{t}\right\})),$$

where NMS represents the non-maximum suppression operation and CAT represents the concatenation operation between the up-to-date detection results and the historical pseudo-label. After updating, {p,t} will be stored in memory and later used as pseudo-labels for unlabeled data.

### 2.4 Spatial-aware Multi-Resolution Feature Extraction

#### 2.4.1 Spatial-aware attention

**Figure 6:**
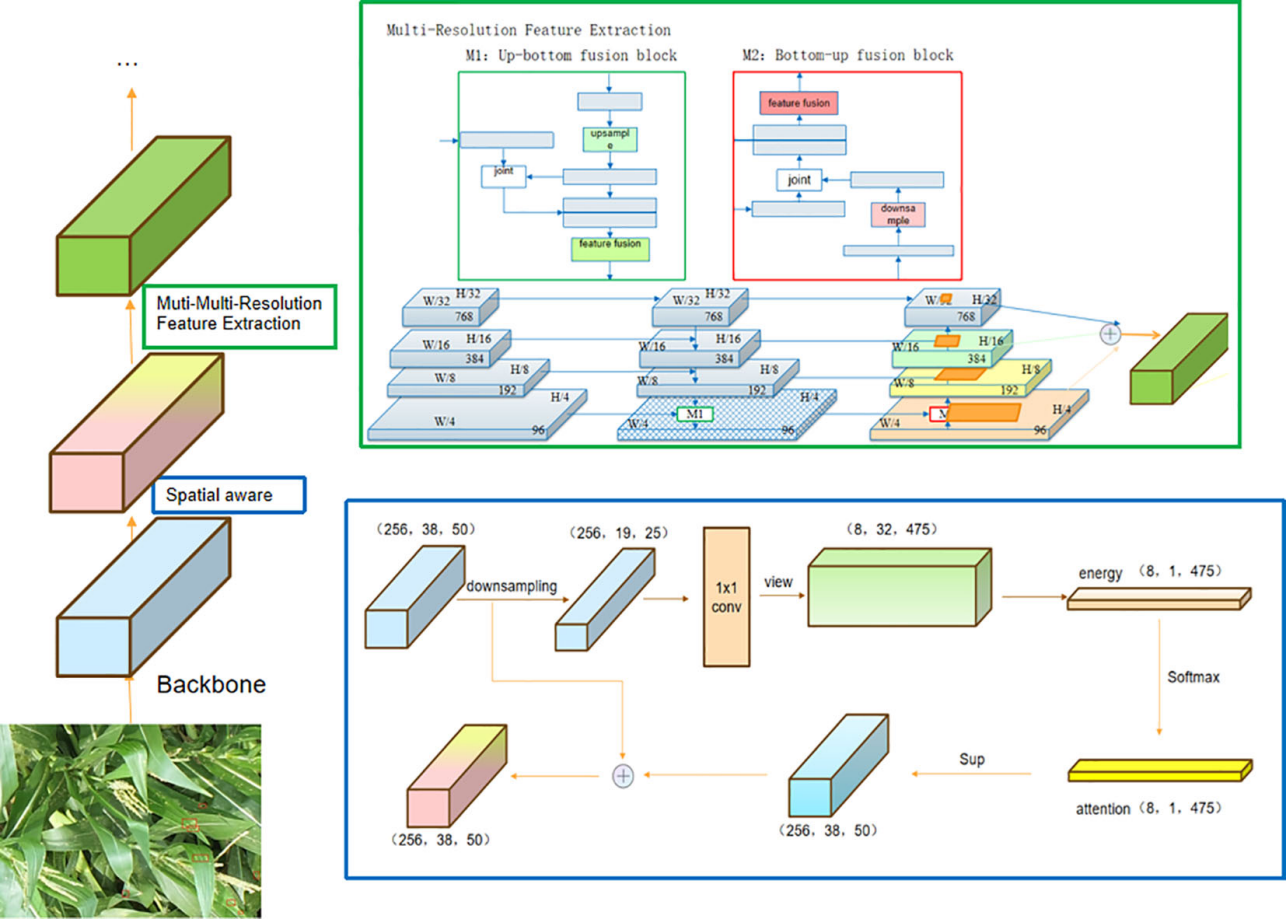
Spatial-aware Multi-Resolution Feature Extraction. The feature map is resolved into spatial-aware attention module and multi-resolution feature extraction module to obtain the optimized features.

It has been recognized that convolutional neural networks have limitations in effectively learning spatial transformations present in images (Liu et al., 2016). Some works mitigate this problem by either increasing the model capability (size) (Krizhevsky et al., 2012) or involving expensive data augmentations (Ghiasi and Fowlkes, 2016), consequently leading to a significant rise in computational expenses during both inference and training processes. Subsequently, novel convolution operators were proposed to improve the learning of spatial transformations. Cheng et al. (2016) proposed to use dilated convolutions to aggregate contextual information from the exponentially expanded receptive field. Chen et al. (2016) proposed a deformable convolution to sample spatial locations by incorporating self-learned offsets. In this study, we introduce a spatial-aware attention mechanism that not only applies attention to individual spatial locations but also adaptively combines multiple feature levels to learn a more discriminative representation. As shown in **Figure 6**, the operation of the spatial-aware module can be outlined in two steps:

1) Query and Key Computation: The values *x_q_* and *x_kv_* are derived through a downsampling operation, serving as inputs for the computation of the Query and Key, respectively.
2) Softmax and Weighted Average: Apply the softmax function to the attention scores and utilize the resulting weights to perform a weighted average on the values, yielding the output.
3) Upsampling and Fusion: Apply upsampling to restore the feature matrix to its original shape and fuse it with the initial features.

Query and Key in the attention mechanism are two important concepts used in the Transformer model to calculate the attention weight. In essence, this spatial-aware mechanism prioritizes the weighting of the Key by utilizing a predefined apprBias. This is followed by the calculation of the Key and Query to obtain the attention score. The final output is obtained through a sequence of operations, culminating in residual concatenation.

#### 2.4.2 Multi-Resolution Feature Extraction

Many studies have emphasized the importance of scale in object detection, as objects with vastly different scales often co-exist in natural images. Early works have demonstrated the significance of leveraging image pyramid methods (Gidaris and Komodakis, 2015; Huang et al., 2019; Wiegreffe and Pinter, 2019) for multi-scale training. However, to enhance efficiency, the concept of a feature pyramid (Ren et al., 2015) was proposed. This approach involves concatenating a pyramid of downsampled convolution features and has since become a standard component in modern object detectors.

As convolutional neural networks become deeper, the repeated downsampling operations lead to the loss of information related to small and overlapping targets. Therefore, shallow-layer features are better suited for localizing small targets, while deep-layer features are more suitable for classifying larger targets, as the receptive field of anchor boxes expands with network depth. To tackle the multiscale challenge posed by target sizes, it has become common practice to employ features of different resolutions. These features are responsible for predicting targets of various sizes, leading to the proposal of a Multi-Resolution Feature Extraction module.

As shown in **Figure 6**, the operation of the Multi-Resolution Feature Extraction module can be outlined in two steps:

1) Up-bottom Path Augmentation: Complete an up-resolution feature fusion. First, change the channel of the input feature and then up-resolution, perform nearest neighbor upsampling on the above features, and then perform feature fusion.
2) Bottom-up Path Augmentation: Complete a reduced-resolution feature fusion. First, change the channel of the input features and then perform downsampling and feature fusion.
3) Adaptive feature pooling: Analyze the ratio of features pooled from different levels with adaptive feature pooling. We use the max operation to fuse features from different levels, which lets the network select element-wise useful information. Specifically, for every candidate region, we associate it with various feature levels, exemplified by the dark-gray region in **Figure 6**. We employ ROIAlign to pool the feature grids from these diverse levels, followed by a fusion operation (pixel-by-pixel SUM or ADD) to amalgamate the feature meshes originating from different levels.

### 2.5 Cascade RPN

High-performing object detectors, such as Faster R-CNN (Ren et al., 2015), adopt a two-stage pipeline approach to tackle the detection problem. To create a sparse set of proposal boxes, an RPN first fine-tunes and prunes a set of anchors. The RPN’s suggestions are then further refined and categorized in the second stage by a region-wise CNN detector (R-CNN). Region proposals are essential in enabling the detector to produce precise bounding boxes while maintaining computational viability. Grouping super-pixels (e.g., Selective Search (Uijlings et al., 2013), Constrained Parametric Min-Cut problems (CPMC) (Carreira and Sminchisescu, 2012), Multiscale Combinatorial Grouping (MCG) (Arbeláez et al., 2014), and window scoring [e.g., objectness in windows (Alexe et al., 2012) and EdgeBoxes (Zitnick and Dollár, 2014)] are the foundation of early approaches for region proposal generation. Despite being the industry standard for object detection in classical computer vision, these techniques have drawbacks because they operate as separate modules from the detector and cannot be computationally efficient.

Numerous studies have been conducted to enhance the performance of the RPN (Gidaris and Komodakis, 2016; Yang et al., 2016; Wang et al., 2019; Zhong et al., 2020). Multi-stage refinement, in which the output of one step is used as the input for the subsequent stage, is the general tendency. As shown in Wang et al. (2019), this iterative method works until accurate localization is attained.

**Figure 7:**
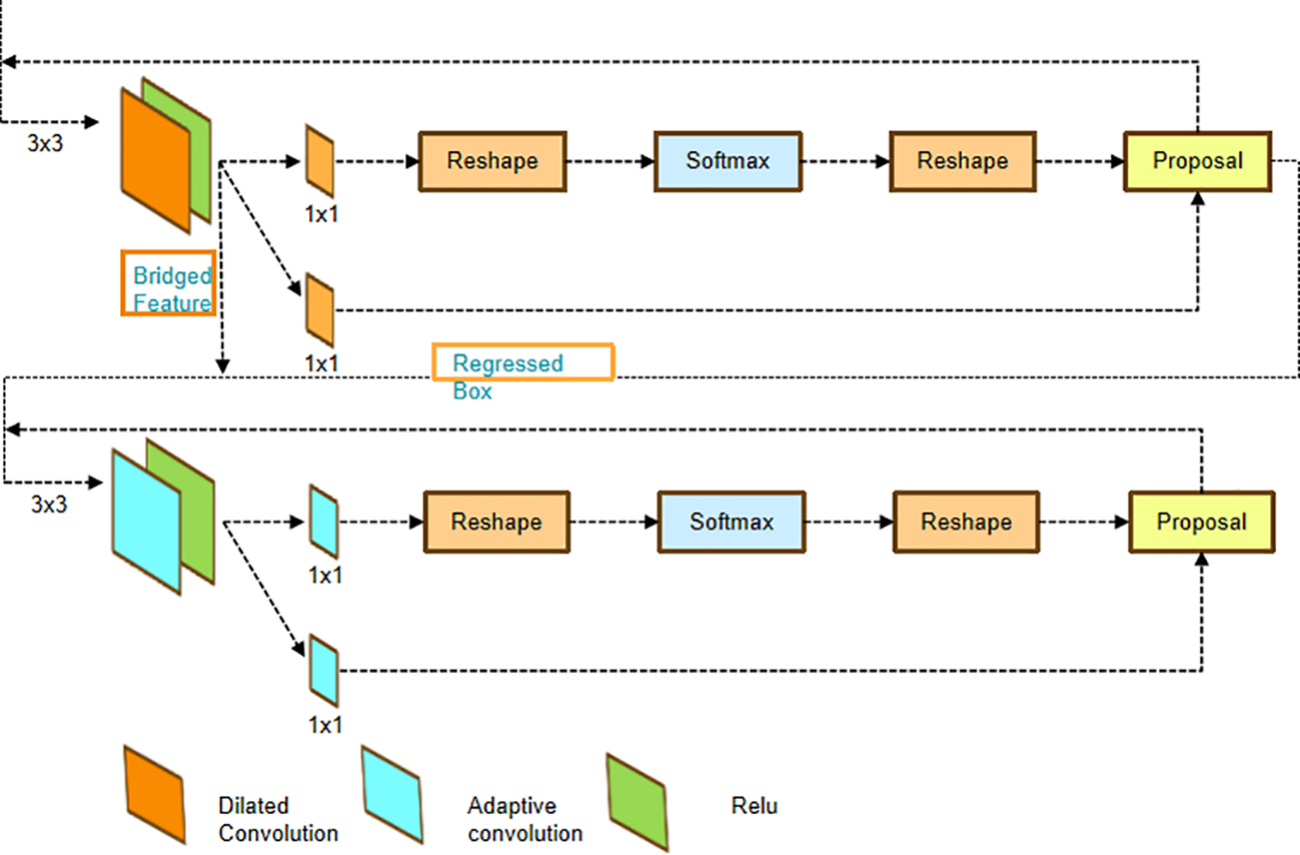
The architectures of Cascade RPN networks. The features of the first stage are “bridged” to the next stages since the spatial order of the features is maintained by the dilated convolution. RPN, Region Proposal Network.

The above method achieved good results in various semi-object detection tasks; however, there are challenges in this study, such as small targets, complex background, and scale change. Compared to objects of typical size, small targets such as pests have the problem that the regressed boxes are more easily misaligned to the image features, breaking the alignment rule required for object detection. It occurs when the anchors, following regression, undergo significant changes relative to their original positions. However, both classification and regression still employ features from the original positions for prediction. To address this concern, we introduce a Cascade RPN module. The architecture of the Cascade RPN is illustrated in **Figure 7**. Cascade RPN uses adaptive convolution to fine-tune the anchor of each stage. The adaptive convolution can be regarded as a lightweight RoI Align layer. Since anchor center offsets are zero, adaptive convolution is used in the first step to achieve dilated convolution. The first stage’s features are then “bridged” to the latter stages, guaranteeing that the dilated convolution preserves the features’ spatial order.

In this module, we use adaptive convolution to ensure alignment between the features and the anchors at every level. This approach takes both the anchors and the image features as inputs and learns the sampled features guided by the anchors.

Given a feature map *x*, in the standard 2D convolution, the feature map is first sampled using a regular grid $\mathbb{R}=\;\left\{\left(r_{x},r_{y}\right)\right\}$ , and the samples are summed up with the weight *w*. Here, the grid ℝ is defined by the kernel size and dilation. For example, ℝ={(−1,−1),(−1,0),(−1,1),(0,−1),(0,0),(0,1),(1,−1),(1,0),(1,1)} corresponds to kernel size 3 × 3 and dilation 1. For each location p on the output feature y , we have Equation 3


(3) $$y\left[p\right]=\sum_{r\in \mathbb{R}}w\left[r\right]\cdot x\left[p+r\right]$$

In adaptive convolution, we have Equation 4, the regular grid ℝ is replaced by the offset field O that is directly inferred from the input anchor.


(4) $$y\left[p\right]=\sum_{o\in O}w\left[o\right]\cdot x\left[p+o\right]$$

Let $\bar{a}$ denote the projection of anchor a onto the feature map. The offset o can be decoupled into center offset and shape offset. The offset o can be formulated as Equation 5


(5) $$o=o_{\text{ctr}}+o_{\text{shp}},$$

where $o_{ctr}=\;\left({\bar{a}}_{x}-p_{x},{\bar{a}}_{y}-p_{y}\right)$ and $o_{shp}$ is defined by the anchor shape and kernel size. For example, if kernel size is 3 × 3, then ***o***_shp_ $\in \left\{\left(-\frac{{\bar{a}}_{w}}{2},\frac{{\bar{a}}_{h}}{2}\right),\;(-\frac{{\bar{a}}_{w}}{2},0),\ldots ,\;(0,\frac{{\bar{a}}_{h}}{2}),\;(\frac{{\bar{a}}_{w}}{2},\frac{{\bar{a}}_{h}}{2})\right\}$ . As the offsets are typically fractional, sampling is performed with bilinear interpolation analogous to Dai et al. (2017).

## 3 Results and discussion

### 3.1 Model evaluation metrics

The measures Precision, recall, Average Precision (*AP*), and mean Average Precision (*mAP*) are frequently used in the object detection task to assess the model’s accuracy. The precise calculation formula can be formulated as Equations 6–11.


(6) $$\text{Precision }=\frac{\#TP}{\#TP+\#FP}$$

(7) $$\text{Recall }=\frac{\#TP}{\#TP+\#FN}$$

(8) $$AP\;=\int_{0}^{1}\text{Precision }d\text{ Recall}$$

(9) $$AP_{50:95}=\frac{1}{10}\left(AP_{50}+AP_{55}+\ldots +AP_{90}+AP_{95}\right)$$

(10) $$mAP_{@0.5}=\frac{1}{C}\sum_{i=1}^{C}AP^{i}$$

(11) $$mAP_{@\left[0.5:0.95\right]}=\frac{1}{C}\sum_{i=1}^{C}AP_{50:95}^{i}$$

where the number of pest targets that are correctly recognized is denoted by *TP* (true positive), the number of pest targets that are mistakenly detected is denoted by *FP* (false positive), and the number of missed pest targets is represented by *FN* (false negative). *C* is the number of pest categories; there are 24 in the Pest24 dataset compared to 1 in the corn borer dataset. The area under the Precision–Recall curve for each pest category in the detection is represented by $AP^{i}$ , the AP for the *i*th category. $mAP_{@0.5}$ is the average of the AP for all pest categories when the IoU threshold is 0.5. $AP_{50:95}$ is the average of the 10 values of *AP*_50_, $AP_{55},\ldots ,AP_{90},AP_{95}$ . $mAP_{@\left[0.5:0.95\right]}$ is the average mAP under different IoU thresholds. $mAP_{@\left[0.5:0.95\right]}$ plays a critical role in evaluating object detection models, offering valuable insights into their ability to strike a balance between recall (detecting objects) and precision (accurately detecting objects) at different levels of object overlap with the ground truth. Thus, $mAP_{@0.5},mAP_{@0.75},mAP_{@\left[0.5:0.95\right]}$ are typically chosen as the primary evaluation metrics in agricultural pest detection jobs in order to provide a more thorough and equitable assessment of pest detection model performance.

### 3.2 Implementation details

We implemented our dataset and network structure code on the open-source platform MMDetection (Chen et al., 2019). Using pre-trained model weights from ImageNet (Deng et al., 2009), we were able to accelerate our training process. Additionally, we applied a consistent image pre-processing process to all comparison networks, which included the following:

1) RandomResize: Randomly change the image size.
2) RandomCrop: Randomly crop the image size.
3) RandomFlip: Randomly flip images and their annotations.
4) RandomErasing: Randomly remove a randomly selected rectangular region with a variable size and aspect ratio.
5) Normalize: Normalize the current image.
6) Padding: Pad the image to the specified size.

Two 32G RAM NVIDIA Tesla V100 GPUs were used for all studies. Pytorch, Python 3.8, and Ubuntu 18.04 comprise the software environment. NVIDIA CUDA10.2 and CUDNN7.6.5 neural network packages were utilized to speed up the training process. The experiment environment listed in **Table 2** was used.

**Table 2:** Experiment environment.

Configuration	Parameter
CPU	Intel Xeon Gold 522
GPU	NVIDIA Tesla V100
Operating system	Ubuntu 18.04
Accelerated environment	CUDA10.2 CUDNN7.6.5

### 3.3 Comparison with other advanced detectors

We have extensively referenced numerous outstanding works and compared our results with them to demonstrate the superior accuracy of our network model. These works include the one-stage algorithm YOLOv5 (Jocher et al., 2021), the two-stage algorithm Faster R-CNN, the anchor-free algorithm CornerNet (Law and Deng, 2018), and the classical semi-supervised object detection algorithms STAC and SoftTeacher. You Only Look Once (YOLO) was originally proposed by Joseph Redmon and others. It is a real-time target detection algorithm. SoftTeacher is an end-to-end pseudo-label-based semi-supervised target detection framework proposed by Mengde Xu and others (Sohn et al., 2020). It is important to note that the following comparison results are provided for reference purposes only. Variations in preprocessing and hardware conditions among different works prevent a strict reflection of the strengths and weaknesses of various methods. The algorithms were executed in an identical experimental environment, with parameters consistent with the original models.

Mainstream semi-supervised target detection algorithms use 1%, 2%, 5%, and 10% training data division. However, the less the amount of annotated data used, the worse the performance of the model. If too much annotated data are used, the semi-supervised algorithm lose their meaning. Considering the amount of annotated data and model performance, we use 20% annotated data to achieve a similar effect to the model trained based on the entire annotated dataset.

As shown in **Table 3**, Faster R-CNN, YOLOv5, and CornerNet are all target detection algorithms based on supervised learning, using 100% No. train. Semi-supervised target detection algorithm STAC SoftTeacher and PestTeacher both use 20% No. train.

**Table 3:** Dataset details.

Dataset	100% No. train	20% No. train	No. val	Resolution
Corn borer	3,950	790	494	608 × 608
Pest24	12,702	2,540	5,075	800 × 600

The quantitative comparison results are presented in **Table 4**. It is evident that the enhanced models have substantially improved the performance of pest region detection. From the concrete evaluation metrics, PestTeacher obtains *mAP*_@0_._5_ 62.1% and 48.9% on the corn borer dataset and Pest24 dataset, respectively. When compared to SoftTeacher and STAC, PestTeacher achieves improvements of 7.3% and 12.3%, respectively. The *mAP*_@[0_._5:0_._95]_ calculated at higher thresholds demonstrates how the recommended approaches support the production of bounding boxes of superior quality. PestTeacher enhanced the *mAP*_@[0_._5:0_._95]_ by 1.2% and 2.8% on the corn borer and Pest24 datasets, respectively. In addition, PestTeacher achieves 79.5% and 74.8% efficacy when compared to the best supervised learning-based detectors, using only 20% of the training set supervised on the Pest24 and corn borer datasets, respectively.

**Table 4:** Comparison of pest detection results between different models.

Method	Backbone	Dataset	mAP_@0.5:0.95_	mAP_@0.5_	mAP_@0.75_
Faster R-CNN	ResNet50	Corn borer	31.3	72.4	16.9
YOLOv5	DarkNet53	Corn borer	32.5	78.1	17.2
CornerNet	ResNet50	Corn borer	20.2	53.0	7.70
STAC	ResNet50	Corn borer	20.3	50.4	8.6
SoftTeacher	ResNet50	Corn borer	21.9	54.8	9.4
PestTeacher	ResNet50	Corn borer	23.3	62.1	9.4
Faster R-CNN	ResNet50	Pest24	32.6	58.5	32.8
YOLOv5	DarkNet53	Pest24	40.6	65.4	34.3
CornerNet	ResNet50	Pest24	30.1	54.6	31.2
STAC	ResNet50	Pest24	21.7	40.1	20.6
SoftTeacher	ResNet50	Pest24	24.6	44.3	24.5
PestTeacher	ResNet50	Pest24	27.4	48.9	28.2

### 3.4 Visualization of detection results

**Figure 8:**
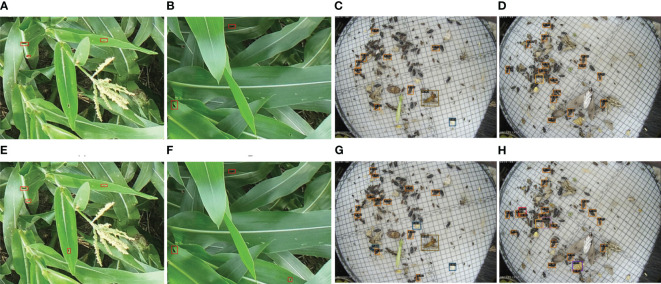
Results of SoftTeacher and PestTeacher algorithms on corn borer and Pest24 datasets.The SoftTeacher model yields detection results denoted as **(A–D)**. Likewise, the PestTeacher model produces detection results labeled as **(E–H)**.

As shown in **Figure 8**, we visually represent a portion of the pest detection findings in this section so that you may see the advantages of our suggested semi-supervised pest detection technique. PestTeacher performs well across several corn growth cycles. Its accuracy rate is higher, and its missed detection rate is lower than that of SoftTeacher; the baseline algorithm is demonstrated in **Figures 8A, B, E, F**. It can be found *via* quantitative and qualitative analyses that the improved model performs well in detecting pests with sparse or dense distribution compared to the original SoftTeacher. As shown in **Figures 8C, G**, where noises (non-target pests with similar appearances) were present in the photos, PestTeacher demonstrated greater robustness since the attention module highlighted the pest traits that were successful and eliminated other distractions. When images containing a dense distribution of small pests are available, PestTeacher detects more pests and fewer misidentifications, as shown in **Figures 8D, H**.

### 3.5 Ablation study

Our proposed semi-supervised pest detector based on the SoftTeacher model contributes four elements, including the mutual learning with memory (MLM), Spatial-aware Multi-Resolution Feature Extraction (SMFE), and Cascade RPN modules developed. To demonstrate the effectiveness of each module in our method, we performed ablation experiments on corn borer datasets, as shown in **Table 5**. The confirmation bias issue and differences in detection results within the same image that arise during many training iterations can be handled using the MLM module. The MLM module enhanced the *mAP*_@0_._5_ by 1.2% on the corn borer datasets. The SMFE operating on the neck layer decreased the missed detection from overlapping pests and enhanced the *mAP*_@0_._5_ by 1.0%. High-quality anchors can be produced by an RPN module with a cascade architecture, which is crucial for semi-supervised object detection. The Cascade RPN module enhanced the *mAP*_@0_._5_ by 3.5%. The *mAP*_@[0_._5:0_._95]_ computed at higher thresholds shows that the suggested strategies aid in the creation of high-caliber bounding boxes.

**Table 5:** Results of ablation experiments on corn borer dataset.

SoftTeacher	MLM	SMFE	Cascade RPN	mAP_@0.5:0.95_	mAP_@0.5_	mAP_@0.75_
✓				21.9	54.8	9.4
✓	✓			21.7	56.0	9.8
✓		✓		22.0	55.8	9.5
✓			✓	21.9	58.3	9.7
✓	✓	✓	✓	23.3	62.1	9.4

The check mark indicates that the method in the same column has been selected.

MLM, mutual learning with memory; SMFE, Spatial-aware Multi-Resolution Feature Extraction; RPN, Region Proposal Network.

To show the efficiency of each module in our system, we conducted ablation experiments on the Pest24 dataset, as shown in **Table 6**. The MLM module enhanced the *mAP*_@0_._5_ by 1.8% on the Pest24 datasets. The SMFE operating on the neck layer decreased the missed detection from overlapping pests and enhanced the *mAP*_@0_._5_ by 0.8%. The Cascade RPN module enhanced the *mAP*_@0_._5_ by 2.2%. The experimental results yielded compelling evidence to validate the efficacy of modules, significantly improving the accuracy of detection metrics.

**Table 6:** Results of ablation experiments on Pest24 dataset.

SoftTeacher	MLM	SMFE	Cascade RPN	mAP_@0.5:0.95_	mAP_@0.5_	mAP_@0.75_
✓				24.6	44.3	24.5
✓	✓			25.6	46.1	25.6
✓		✓		24.8	45.1	24.6
✓			✓	25.8	46.5	25.9
✓	✓	✓	✓	27.4	48.9	28.2

The checkmark indicates that the technique in the same column was picked.

MLM, mutual learning with memory; SMFE, Spatial-aware Multi-Resolution Feature Extraction; RPN, Region Proposal Network.

## 4 Conclusion

Agricultural pests have become the main factors affecting and restricting grain production due to their high frequency of occurrence, wide occurrence area, and serious harm. In the past, researchers mostly chose to apply excessive and purposeless chemical pesticides to solve pest problems. Although agricultural losses can be reduced to a certain extent, the negative impacts such as pesticide residues and environmental pollution caused by the use of chemical pesticides are becoming increasingly prominent. Therefore, it is particularly important to predict pests and carry out effective and targeted prevention and control. In this case, an important prerequisite for effective pest prediction is the accurate identification and detection of pests.

Although pest detection based on supervised learning has accomplished many achievements in actual agricultural production activities, it relies heavily on a large amount of manual annotation data and requires many manpower and material resources, causing difficulties in practical applications. Detection algorithms based on semi-supervised learning alleviate the problem of data annotation and can achieve results similar to those based on supervised learning algorithms using only a small amount of annotated data.

The paper proposes a PestTeacher, a novel semi-supervised object detection (SSOD) framework that achieves good results in two semi-supervised object detection tasks. PestTeacher effectively mitigates the issues of confirmation bias and instability among detection results across different iterations by mutual learning with memory mechanism. To address the issue of leakage caused by the weak features of pests, we propose the Spatial-aware Multi-Resolution Feature Extraction module. Compared to the baseline model SoftTeacher, our model improves *mAP*_@0_._5_ at 7.3 compared to that of SoftTeacher at 4.6. Furthermore, we introduce a Cascade RPN module to generate higher-quality anchors. Through the above method, PestTeacher achieves better pest detection results than the baseline algorithm. While we assess using the Faster R-CNN two-stage detector, our suggested PestTeacher is not limited to object detection models. This implies that PestTeacher can be immediately used with other detectors, such as the one-stage SSD and FCOS detectors, which we will save for later research.

## Data availability statement

The raw data supporting the conclusions of this article will be made available by the authors, without undue reservation.

## Author contributions

JZ: Writing – original draft, Writing – review & editing. HH: Supervision, Writing – review & editing. YS: Supervision, Writing – review & editing. JC: Software, Writing – original draft. WZ: Validation, Writing – review & editing. FQ: Visualization, Writing – review & editing. HY: Writing – original draft.
